# Peroneal Tendon Dislocation: A Report of Two Cases

**DOI:** 10.7759/cureus.34949

**Published:** 2023-02-13

**Authors:** Mohammed Maroc, Zakaria Khatab, Othman Moueqqit, Najib Abdeljaouad, Hicham Yacoubi

**Affiliations:** 1 Department of Trauma and Orthopaedics, Mohammed VI University Hospital, Faculty of Medicine and Pharmacy, Mohammed First University, Oujda, MAR; 2 General Medicine, Faculty of Medicine and Pharmacy of Oujda, Mohammed First University, Oujda, MAR

**Keywords:** ankle instability, lateral ankle ligament instability, ankle injuries, sport injury, peroneal dislocation

## Abstract

Ankle injuries are a very common cause of patient visits to the primary care units and emergency departments. Although the most frequent ones are lateral ligament sprains, peroneal tendon subluxations have the same inversion mechanism and are described as one of the main causes of lateral ankle pain and instability. They are often missed during the acute phases as they are misdiagnosed as ankle sprains since both injuries share similar mechanisms and often occur in athletes and patients with high sports activity.

We present two different cases of peroneal tendon dislocation that illustrate how this pathological condition may be present under different circumstances. We aim, through these cases, to provide clinical awareness and help improve earlier diagnosis of this condition; we also demonstrate the effectiveness of surgical reattachment of the upper retinaculum that two of our patients underwent.

## Introduction

Ankle injuries are a very common cause of patient visits to the primary care units and emergency departments [1,2]. Although the most frequent ones are lateral ligament sprains, peroneal tendon subluxations have the same inversion mechanism and are described as one of the main causes of lateral ankle pain and instability [1,3]. Its exact prevalence remains unknown. However, it has been estimated that up to 30% of patients who had surgery for ankle instability have been found to have a peroneal tear [4]. Thus, the condition remains underdiagnosed, and awareness should be raised by different clinicians towards this pathologic condition as it is often misdiagnosed with lateral ankle sprains [1,2].

Peroneal tendon dislocation was first described by Monteggia in 1803 in a ballet dancer [2]. It is a very uncommon injury for its both acute and chronic forms [5]. As many cases may be missed during the acute phase, patients may present with chronic instability, ankle pain, ankle stiffness, and snapping [6]. The mechanism of the dislocation is often thought to be violent dorsiflexion or eversion of the foot [2]. It often occurs among soccer and skiing athletes but may also be associated with calcaneal fractures and some neurological conditions such as poliomyelitis [2]. Magnetic resonance imaging is the best way to establish the diagnosis, but a proper history and clinical assessment are mandatory [1,2]. Although most authors confirm that early diagnosis and surgical repair of the injury remain the treatment of choice, different surgical techniques are still discussed in the literature for the chronic form [5].

In light of this, we present two different cases of peroneal tendon dislocation that illustrate how this pathological condition may be present under different circumstances. We aim, through these cases, to provide clinical awareness and help improve earlier diagnosis of this condition; we also demonstrate the effectiveness of surgical reattachment of the upper retinaculum (UR) that two of our patients underwent.

## Case presentation

Case 1

A 36-year-old policeman with no prior medical history was involved in a road traffic accident when his motorcycle was hit by a car, which directly impacted his left ankle. He was initially treated for an ankle sprain and immobilized with an ankle orthosis for 40 days. However, three months later, he presented with ongoing lateral ankle instability that occurred during dorsiflexion or eversion of the foot, despite the treatment. The patient also complained of pain (6/10 visual analog score) and ankle popping. On physical examination, his vitals were stable. A positive Sobel test (peroneal tunnel compression test) found a peroneal tendon dislocation. The rest of the physical examination had no other abnormal signs. Further work-up consisted of X-ray and MRI studies of the ankle, both revealing no abnormalities.

**Figure 1:**
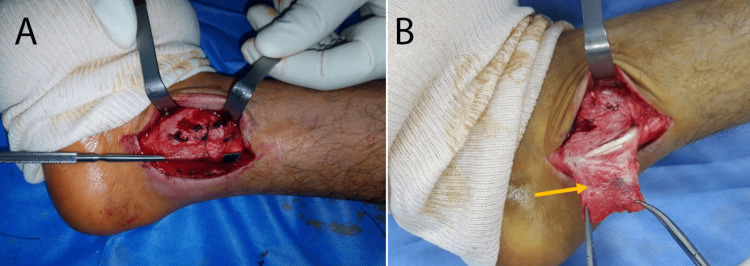
A: View of the final set-up of the superior peroneal retinaculum repair, B: Superior peroneal retinaculum (orange arrow) before repair

Surgery was performed with the patient laying on his back using local anesthesia. Cutaneous and subcutaneous incisions were made, revealing fibular sheath detachment classified as stage 1 according to Eckert and Davis classification [7]. The dislocation was easily reproducible by manual maneuvering of the foot. The decision was to go for anatomic reattachment of the retinaculum (ARUR). The detachment was closed by three anterior trans-osseous sutures after peroneal tendon reduction (Figure 1). Reconstruction of the superior retinaculum was performed using a periosteal flap from the lateral malleolus.

The patient benefited from a windowed plaster cast boot for 45 days with prolonged physical rehabilitation. Full foot support was obtained two months after the surgery, with no reported complications.

Case 2

A 25-year-old male medical student with no significant past medical history presented to our department for ankle instability for two months after he had been a victim of a sports accident. He received a direct kick on the outside of his left ankle while playing soccer. The patient reported experiencing up to five ankle sprains per day during the two months period after his accident. He was able to recognize the peroneal dislocation and manually reduced it in multiple instances. He confirmed the incident was triggered by dorsiflexion and eversion of the foot. However, he reported no pain or ankle popping. His vital signs were stable. Physical examination showed peroneal tendon dislocation with a positive Sobel test. The rest of the physical examination had no other abnormal signs. Further work-up consisted of an X-ray and MRI study of the ankle, both revealing no abnormalities.

**Figure 2:**
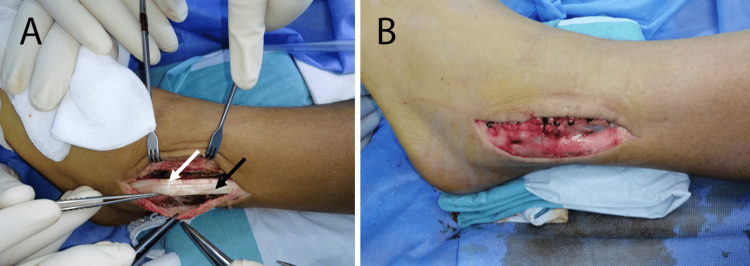
A: View of the peroneal tendons: peroneus longus (black arrow) and peroneus brevis (white arrow). B: View of the final set-up of the superior peroneal retinaculum repair

Surgery was performed using the same procedures described in case 1 (Figure 2). According to Eckert and Davis's classification, the incision revealed a fibular sheath detachment classified as stage 1 [7]. A posterior foot splint was applied for 15 days, followed by circular plaster for 30 days. The patient underwent prolonged physical rehabilitation for 45 days and he obtained full foot support after two months; he could not return to sports activity for 18 months. Our patient reported chronic pain in his ankle (triggered mainly by a cold) after the surgery.

## Discussion

Peroneal tendon dislocation is a very rare condition [8]. According to numbers shared by Espinosa and colleagues in 2015, among more than 23,000 ankle injuries that occur every day in the USA, less than 0.5% are diagnosed as peroneal subluxations and dislocations [2]. The very low incidence may also be due to frequent misdiagnoses as ankle sprains [8,9]. The main cause of the injury is sports accidents [10,11] and the main described mechanism is sudden dorsiflexion and eversion of the foot with forced contraction of fibular muscles [2,10]. According to the Eckert-Davis classification system, the injury can be graded on a scale of 1 to 3 [4]. In our study, both of our patients had a grade 1 dislocation as the retinaculum with the periosteum was stripped off the lateral malleolus, according to the same system.

Anatomically, the two peroneal tendons (the peroneus longus and peroneus brevis) share the common peroneal sheath, as it travels through the superior peroneal tunnel formed by the peroneal retinaculum with the fibular groove being an osseous floor and the posterior intermuscular septum of the leg as the non-osseous floor of the same tunnel [4,12]. Hence, the morphology and the integrity of the malleolar groove and the superior peroneal retinaculum (SPR) have the most influence on peroneal tendons injuries since the peroneal muscles are the first muscles to react when a sudden ankle inversion takes place [4,12,13]. A delayed response of these muscles following sudden inversions of the foot during ankle sprains leads to functional ankle instability [12].

Patients with chronic peroneal tendon dislocation or subluxation often present with chronic ankle pain, ankle instability, ankle stiffness, and snapping [6]. For our patients, ankle instability and pain were the main concerns. Both of our patients underwent non-operative treatment before having chronic ankle instability. Thus, reporting these two cases comes with a reminder for every emergency or orthopedic clinician to keep awareness of this pathological condition in different ankle injuries, especially in athletes.

Clinically, the diagnosis of peroneal dislocations may be a challenge due to the similarity of the mechanisms between this entity and other frequent ankle injuries [1,2,5,11]. However, conventional radiography, ultrasound, MRI, and CT scan are all known to help elucidate the integrity of the peroneal tendons and diagnose injuries [1,2,13]. While radiography may help detect bone injuries, MRI is better at assessing the integrity of the peroneal tendons as well as the integrity of the SPR [1-6,8,10]. Unfortunately, in both our cases, MRI was inconclusive and it can be considered one of the limitations of this study.

Following acute phases of peroneal tendon dislocations, the conservative treatment seems to be the first choice [11,14]. However, the dislocation’s reoccurrence rate may be as high as 60% [11,13]. Operative management is the choice for recurrent dislocations but also for athletes during the acute phase [11,13,14]. To date, although the literature has not yet established the best surgical approach [15], different operative techniques have been described such as bone block procedures and the reattachment of the SPR with or without groove deepening [13]. While all these surgical techniques are performed under direct vision, some tendoscopic surgical techniques have been also described. According to a study assessed in July 2022 [11], tendoscopic procedures are relatively complicated and use suture anchors and not tying elements which may cause subcutaneous irritation [11]. The study introduced a knotless tendoscopic peroneal retinaculum repair technique using a Knotless FiberTak (NFT) (Arthrex, Naples, FL), a technique that has not yet been reported [11]. The technique comes with disadvantages as well as it is technically demanding, takes longer operative time, and is not indicated for patients with bone fragility as the NFT maneuvering requires strong pulling of the shuttling sutures [11].

The different surgical techniques seem to be validated according to the literature regarding clinical and functional outcomes [11,13,14-16]. However, the authors are more focused on comparing the time to return to sports after the surgery between the different available techniques [4,13,16,17]. In our study, only the second patient practiced sports regularly before the injury. He underwent simple SPR repair without groove deepening and needed 18 months after the surgery before being able to return to his activity. Compared to literature reviews, the duration is much higher; multiple studies show a duration between two and six months for patients to return to full sports activity [4,13,16,17].

In a comparative retrospective study done by Deng et al., clinical outcomes and return to sports activity have been evaluated between patients who underwent reattachment of the SPR and others who benefited from bone block procedures [13]. The study found results satisfactory with low rates of reoccurrence for both groups, but return to sports activity was faster in cases of reattachment of the SPR with a median time of five months compared to a median of six months for bone block procedure patients [13]. In another comparative study, Tomihara et al. shared similar results as the time to return to sports activity was significantly faster in athletes after reattachment of the SPR with a mean period of 2.9 months than after the bone block procedure (3.9 months) [17]. On the other hand, a similar study by Mercer and colleagues compared clinical outcomes and time of return to sports between patients who underwent SPR repair with and without groove deepening [16]. While results were positive for both groups, return to sport was faster for patients who benefited from SPR repair with groove deepening [16]. For patients with a high sports activity engagement, combining SPR repair with groove deepening comes with the best anatomic and functional stability [4].

## Conclusions

Peroneal tendons subluxation or dislocation is a relatively rare entity that can go undiagnosed during acute phases of ankle injuries. It is often misdiagnosed as simple lateral ankle sprains, which leaves patients with chronic ankle pain and instability. Conventional radiography and MRI help assess the diagnosis and better explore the tendons and the retinaculum integrity. Operative is the best choice for patients with chronic dislocation. The upper peroneal retinaculum repair and bone block procedures are the most common surgical approaches. And while all techniques were validated by the literature to have excellent clinical results, superior retinaculum repair with groove deepening seems to be the best choice for patients with high sports activity.
